# Sensor Fusion-Based Machine Learning Algorithms for Meteorological Conditions Nowcasting in Port Scenarios

**DOI:** 10.3390/s26020448

**Published:** 2026-01-09

**Authors:** Marwan Haruna, Francesco Kotopulos De Angelis, Kaleb Gebremicheal Gebremeskel, Alexandr Tardo, Paolo Pagano

**Affiliations:** 1 Consorzio Nazionale Interuniversitario per le Telecomunicazioni, National Laboratory of Photonic Networks & Technologies (PNTLab), Via Giuseppe Moruzzi, 1, 56124 Pisa, Italy; kaleb.gebremeskel@cnit.it (K.G.G.); alexandr.tardo@cnit.it (A.T.); paolo.pagano@cnit.it (P.P.); 2 TeCIP Institute, Scuola Superiore Sant’Anna, Via G. Moruzzi, 1, 56124 Pisa, Italy

**Keywords:** sensor fusion, autonomous vessels, MASS, meteo nowcasting, machine learning, ports, oneM2M

## Abstract

Modern port operations face increasing challenges from rapidly changing weather and environmental conditions, requiring accurate short-term forecasting to support safe and efficient maritime activities. This study presents a sensor fusion-based machine learning framework for real-time multi-target nowcasting of wind gust speed, sustained wind speed, and wind direction using heterogeneous data collected at the Port of Livorno from February to November 2025. Using an IoT architecture compliant with the oneM2M standard and deployed at the Port of Livorno, CNIT integrated heterogeneous data from environmental sensors (meteorological stations, anemometers) and vessel-mounted LiDAR systems through feature-level fusion to enhance situational awareness, with gust speed treated as the primary safety-critical variable due to its substantial impact on berthing and crane operations. In addition, a comparative performance analysis of Random Forest, XGBoost, LSTM, Temporal Convolutional Network, Ensemble Neural Network, Transformer models, and a Kalman filter was performed. The results show that XGBoost consistently achieved the highest accuracy across all targets, with near-perfect performance in both single-split testing (R^2^ ≈ 0.999) and five-fold cross-validation (mean R^2^ = 0.9976). Ensemble models exhibited greater robustness than deep learning approaches. The proposed multi-target fusion framework demonstrates strong potential for real-time deployment in Maritime Autonomous Surface Ship (MASS) systems and port decision-support platforms, enabling safer manoeuvring and operational continuity under rapidly varying environmental conditions.

## 1. Introduction

The ability to navigate modern maritime ports is becoming increasingly complex due to constant changes in weather, currents, and operational constraints. This situation presents unique challenges to both modern and traditional ships; the need to develop and improve situational awareness is vital to ensuring that port operations are conducted in a safe environment. Nonetheless, improving the situational awareness will inherently improve the efficiency of those operations that, in the “port of the future”, will be undertaken by autonomous ships (MASS) in conjunction with Remote Operation Centres (ROCs) [1].

Most sensors deployed in port environments, such as Light Detection and Ranging (LiDARs), radars, anemometers, weather stations, cameras, and Global Navigation Satellite System (GNSS), provide crucial streams of operational and environmental data. Due to the heterogeneous nature of these data coming from multiple data sources, sensor fusion is needed to combine them. This process greatly improves the reliability, accuracy and completeness of the data. For instance, radar provides strong range coverage, while infrared and optical sensors offer fine angular precision; combining both helps reduce uncertainty and improve the tracking of nearby dynamic entities [2].

While several efforts have explored environmental forecasting and sensor fusion in maritime contexts, most have focused either on open-sea navigation or on single-modality sensing systems, leaving a gap in port-specific, real-time predictive frameworks.

Ports now require integrated solutions that can nowcast localised operational and weather conditions in real time. Having this capability is crucial in the ports of the future, as any variation in environmental conditions must be accounted for by prediction algorithms to ensure the safety of various entities during berthing, docking, and manoeuvring.

In this paper, we propose and validate an IoT-enabled, oneM2M-compliant sensor fusion framework deployed in a private cloud at the Port of Livorno. The framework integrates data from meteorological stations, anemometers, and vessel-mounted LiDARs using feature-level fusion to synchronise multimodal data streams. On top of this infrastructure, we evaluate and compare a suite of machine learning and deep learning algorithms, including Random Forest, XGBoost, LSTM, Temporal Convolutional Network, Ensemble Neural Network, Transformers, and a Kalman Filter, for real-time nowcasting of the three wind targets. The model predicts gust speed, sustained wind speed, and wind direction, which are collectively referred to as wind targets throughout this work. Therefore, the objectives of this work are summarised as follows:

1. To design and validate a hybrid sensor fusion architecture that unifies heterogeneous maritime data sources for environmental nowcasting.
2. To benchmark multiple machine learning models and algorithms for short-term *wind targets* nowcasting in port conditions.
3. To assess the robustness and generalisation of these models under real-world constraints and propose a pathway for integration into future MASS and digital twin systems.

This study contributes to the development of data-centric, AI-assisted situational awareness for smart ports, an essential step toward autonomous maritime operations and smart remote operation centres.

The rest of the paper is organised as follows: Section 2 reviews the existing literature on sensor fusion, machine learning, and environmental prediction within maritime and related domains, highlighting the gaps this work aims to address. Section 3 presents the proposed architecture, detailing the data sources, oneM2M-compliant infrastructure, and the machine learning pipeline used for wind targets nowcasting. Section 4 reports the experimental results and comparative performance of different algorithms under real port conditions. Section 5 provides an in-depth discussion of the findings, including practical implications, observed limitations, and insights for operational deployment. Finally, Section 6 concludes the paper and outlines future research directions, focusing on extending the dataset, refining the models, and exploring broader integration with port digital twins and MASS operations.

## 2. Related Works

The fields of sensor fusion, environmental prediction, and maritime IoT systems have seen rapid advances, yet integration across these domains for port-specific predictive modelling remains limited.

A transformer-based model that deeply integrates RGB, long-wave infrared, LiDAR, X-band radar, and electronic chart data, creating a bird’s-eye view around a vessel, demonstrated robustness with its multimodal integration in adverse conditions during real-world trials [3]. However, this work highlights the value of multimodal fusion for perception, yet its focus is situational awareness in open-sea navigation, not environmental prediction or real-time nowcasting in ports. Another study on sensor fusion proposed a fusion framework integrating radar, AIS, and visual camera data for maritime surveillance; Venice’s ARGOS was used to demonstrate efficacy in detecting and tracking non-cooperative targets in congested waterways [4]. The work extended visual fusion methods for maritime surveillance, showing effectiveness in tracking non-cooperative targets. However, their framework addressed detection rather than prediction, and lacked temporal modelling capabilities essential for forecasting.

A statistical anomaly detection system that predicts system faults based on data from a straddle carrier hydraulic sensor demonstrated high accuracy and an F1 score [5]. However, the study focuses on operational analytics rather than environmental dynamics or sensor fusion-based prediction.

Machine-to-machine (M2M) communication has become an integral part of complex systems; significant work has been done in this area, including the creation of global standards for the common service layer for M2M communication. The oneM2M partnership project is responsible for this task. The players in this industry have been developing commercial and open-source oneM2M-compliant software. Telecom Italia (TIM) described in the AUTOPILOT project [6] the deployment of a oneM2M-compliant IoT platform with REST APIs, SSL security, access control policies, and device provisioning. However, this deployment was applied in a smart-city pilot use case, not maritime or port scenarios. Another test was conducted by researchers on how to scale the oneM2M IoT open-source platform in a software-defined network (SDN) for an optical network controller scenario; this research found that the platform can be used in pre-production network deployments [7]. Nonetheless, this platform was not tested or evaluated in maritime or port scenarios.

In [8], the authors studied a sequential nowcasting framework derived from a process-agnostic Kalman filter, which used constrained regression to estimate signals not directly observed from near real-time data. This work demonstrated a sensor-fusion framework using health data, rather than the maritime or port domain.

Ref. [9] used data from the Automatic Identification System (AIS) to train two sets of artificial neural networks (ANN); they first trained a two-class network and a three-class network. Their models can detect AIS-related anomalies with around 99.9 per cent accuracy, in the order of microseconds. This type of fusion at the model level is a decision-level (late) fusion and focuses only on AIS data.

Using historical data from the port of Bordeaux, a machine learning-based application was trained to predict vessel turnaround time; this system outperforms the manual expert-based system [10]. Nonetheless, this study was confined to measuring timing metrics rather than to environmental prediction or sensor-fusion nowcasting.

Another paper uses an autoencoder to detect anomalies in time-series data streams [11]; it first applies a band-pass filter to reduce noise, then uses a functional neural network autoencoder to detect anomalies. This paper’s approach was generic and did not perform sensor fusion or real-time forecasting.

## 3. Materials and Methods

In this section, we will lay out the details of the architecture, datasets, and the techniques employed to overcome the limitations discussed in the previous chapters. First, we will describe the sensors present in the port and on the ship. Next, we will describe the IoT-enabled, oneM2M-compliant infrastructure we use to integrate heterogeneous environmental and operational sensor data. After this, we will discuss the preprocessing steps we applied to ensure quality and consistency across modalities, such as LiDAR and meteorological sensors. In addition, we will present the machine learning pipeline for real-time nowcasting, the choice of models, the training strategy, and the evaluation metrics. Lastly, the experimental setup and simulation environment were used to assess the system’s performance under representative port conditions.

### 3.1. Data Sources

Maritime operations in a port environment depend highly on various data sources; we have obtained them from both fixed environmental sensors deployed across the port area and from operational sensors installed on board a test vessel. The environmental sensors continuously provide meteorological, hydrodynamic, and atmospheric conditions, while the operational sensors continuously capture the ship’s immediate navigational context. Combining these heterogeneous data streams represents the basis of our sensor fusion and predictive modelling. In the present study, the dataset’s temporal extent (approximately 10 months) reflects an inherent characteristic of the oneM2M-compliant architecture adopted at the Port of Livorno. Unlike traditional, centralised forecasting infrastructures that accumulate multi-year archives, our system primarily functions as a store-and-share platform, designed to ensure interoperability, real-time accessibility, and efficient data dissemination across heterogeneous maritime services. As a consequence, long-term historical storage is not the platform’s current operational objective. Instead, it prioritises the timely ingestion, synchronisation, and sharing of sensor streams for immediate decision support. This architectural philosophy inevitably constrains the volume of historical data available for building predictive models. Despite this limitation, evaluating nowcasting models under reduced data availability is not only necessary but also methodologically valuable. Real-world port deployments often encounter scenarios where only short-term sensor histories are available, due to system maintenance, sensor replacement, storage policies, or the early phases of infrastructure rollout. Ensuring that forecasting models remain functional, stable, and accurate under such constraints is therefore crucial for practical applicability. By designing and testing our models on a limited yet realistic dataset, we explicitly assess the system’s ability to generalise in conditions where deep historical archives are not guaranteed. Moreover, short-term datasets offer an opportunity to assess the robustness of different model classes under restricted temporal dynamics. While large-scale deep learning architectures typically rely on extensive historical patterns, operational decision-making in port environments often requires models that can rapidly adapt, capture short-range dependencies, and remain effective with sparse data. The decision to rely on a time-limited dataset is driven by both the realistic operational constraints of the oneM2M store-and-share pipeline and careful methodological considerations. Testing the models under limited-data conditions ensures that the proposed nowcasting framework remains deployable, scalable, and reliable in real port environments, where extensive historical continuity cannot always be assumed.

For this work, we selected a subset of these sensors: for the environmental sensors category, we selected data from meteorological stations and anemometers, while for the operational sensors, we used LiDARs installed on the ship.

#### 3.1.1. Environmental Sensors

**Figure 1:**
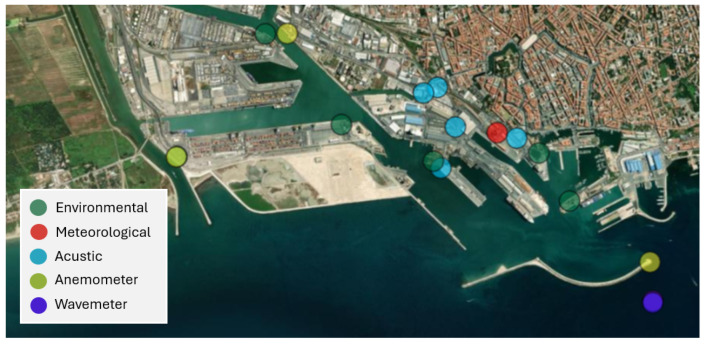
Sensors at the port of Livorno.

The sensors previously mentioned serve as the backbone of our port monitoring system. Among them, the meteorological station measures temperature, pressure, wind speed, wind direction, dew point, and more; all these variables influence local weather dynamics. The anemometer, on the other hand, complements these measurements by providing high-resolution measurements such as wind speed, wind direction, humidity, pressure, etc. The Figure 1 shows the placement of the heterogeneous IoT sensors within the seaport area. The graphical visualisation is provided by a web application used by the Port Authority of Livorno for real-time monitoring, as described in [12].

#### 3.1.2. Operational Sensors

**Figure 2:**
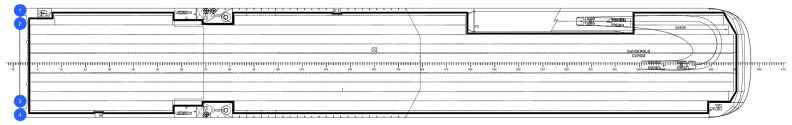
Placement of the LiDARs on the ship.

The operational sensors used in this study are mounted on a vessel to enhance situational awareness during navigation and port manoeuvres. Data from the LiDARs enhances the ship’s object detection capabilities, including in low-light conditions, by providing three-dimensional spatial mapping and precise distance measurements. The Figure 2 shows the position of the LiDARs (blue dots on the schema) installed on board the ship. The LiDARs are pointed at different angles to ensure full visibility of the dock during the berthing operations. The raw data acquired from the LiDARs are retrieved via an on-board 5G Consumer Premises Equipment (CPE) that leverages the existing 5G infrastructure at the Port of Livorno. The fusion of these data improves the prediction and situational awareness capabilities of our nowcasting model.

### 3.2. System Architecture

The system architecture used in this study follows the three-canonical-layer approach and integrates heterogeneous port and vessel data sources into a unified private cloud environment [12]. This architectural design was chosen specifically because it offers interoperability, real-time response, and scalability for sensor-fusion-driven predictive analytics.

The physical infrastructure layer is divided into an IoT layer, which comprises all sensors that collect measurements from the surrounding environment, and a connectivity layer that enables connectivity between the physical layer and the private cloud via various communication channels (e.g., 4G/5G, fibre optics). The oneM2M platform, along with various data sources, forms a data lake accessible through the Data Virtualisation Layer (DVL) component. Thanks to the DVL, it is possible to virtualise data and abstract it from its actual representation. On top of this infrastructure, there is the Application layer, which includes smart applications that provide maritime services to port community end users (e.g., Port Authority, Container Terminals, Coast Guard, etc.).

#### 3.2.1. oneM2M-Compliant IoT Platform

The IoT middleware adopted in this work is based on the oneM2M standard, a global machine-to-machine (M2M) and IoT communication framework [13] jointly developed by a consortium of standardisation bodies including ETSI. The goal of oneM2M is to provide a common service layer that sits between IoT devices and applications, enabling interoperability, scalability, and uniform data management independent of underlying communication technologies. In practical terms, the standard defines a set of common service functions, such as device registration, data storage, discovery, subscription, security, and access control, that ensure heterogeneous sensors and applications can interact seamlessly within a unified architecture. The oneM2M ecosystem is organised around a hierarchical resource tree, where every data element, sensor stream, and application entity is represented as a standardised resource with a unique identifier and a set of attributes. This abstraction makes it possible to harmonise data from different vendors and protocols while preserving temporal alignment and semantic consistency. Additionally, oneM2M supports RESTful APIs (HTTP, CoAP, MQTT), which simplifies integration with cloud platforms, data analytics pipelines, and smart-port services. To implement this standard, our system employs Mobius [14], an open-source oneM2M-compliant platform developed by the Korea Electronics Technology Institute (KETI). Mobius acts as the Common Service Entity (CSE) of the architecture, providing the core service functions defined by the standard (e.g., data ingestion and persistence, resource management, device and application registration, subscription and notification mechanisms, and access control). Mobius exposes RESTful endpoints that allow both port-side environmental sensors (e.g., meteorological stations, anemometers) and ship-borne operational sensors (e.g., LiDAR streams) to publish their data in a uniform and synchronised manner. As the central middleware, it creates a virtualised data layer where raw IoT streams are stored, tagged, and made accessible to higher-level applications, including the sensor-fusion and nowcasting modules developed in this work. This oneM2M-compliant deployment is particularly advantageous in maritime environments, where sensor ecosystems are inherently heterogeneous and long-term data archiving is not always the primary operational requirement.

#### 3.2.2. Data Flow Pipeline

The data flow pipeline in our architecture is designed primarily to support the execution of machine learning and AI-driven nowcasting algorithms, rather than to simply transport sensor measurements across the system. While the heterogeneous IoT infrastructure ensures that environmental and operational measurements reach the private cloud-based environment, the central objective of the pipeline is to transform these raw data streams into a temporally aligned, quality-controlled, and feature-rich dataset that can be directly consumed by our nowcasting models. At the ingestion stage, environmental sensors (e.g., meteorological stations and anemometers) and LiDAR-based operational data streams are published to the oneM2M-compliant platform (Mobius), where they are immediately enriched with metadata, temporally indexed, and made accessible through standardised APIs. These steps ensure semantic and temporal coherence, which is essential for downstream AI tasks that require consistent sample intervals and synchronised modalities. The data pipeline, therefore, acts as a continuous feed to the AI models, providing preprocessed multimodal data that the models use to nowcast short-term wind targets. This design ensures that the emphasis is on analytics and decision-making logic rather than on data transport, and that the forecasting models remain tightly integrated with the underlying IoT ecosystem.

**Figure 3:**
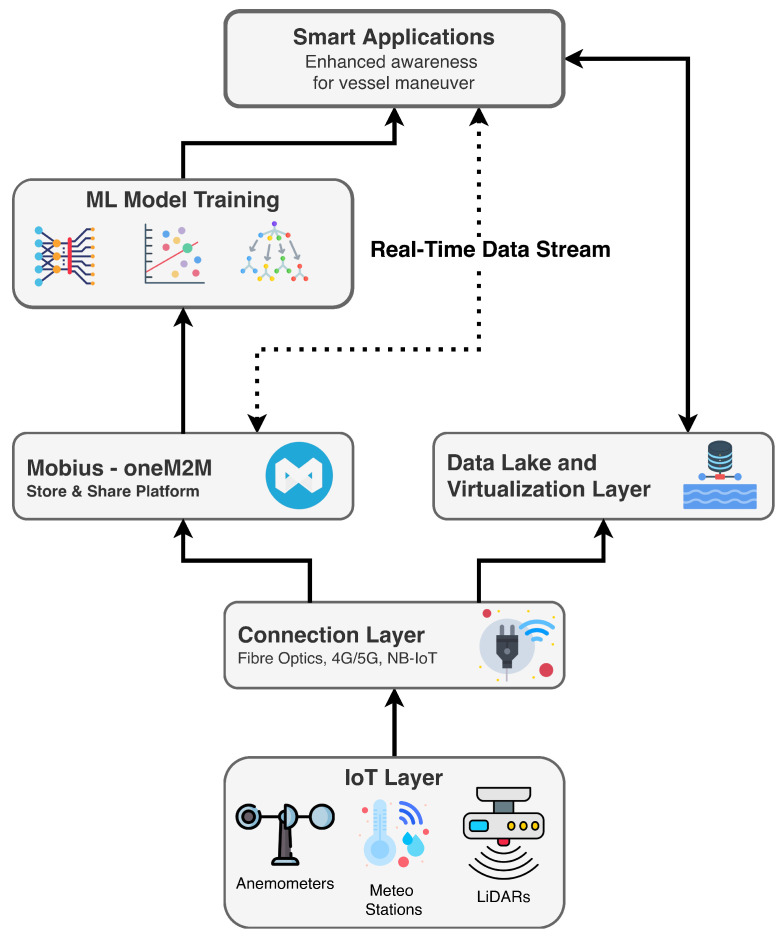
Data Flow Pipeline.

Furthermore, in the data lake sublayer, the data ingested into the Mobius platform can be easily accessed via HTTP-based requests from the Data Virtualisation Layer (DVL) in conjunction with the underlying message broker from the service management and orchestration sublayer. This flow allows developers to access data from multiple sensors in a harmonised way and ensures interoperability among sensors. Finally, smart applications can use the data for predictive analysis. The enhanced awareness of vessel manoeuvre application can utilise this data pipeline to analyse meteo-marine conditions and adjust the manoeuvre planning accordingly. Using such an end-to-end pipeline ensures that the system can perform sensor fusion and run predictive algorithms on consistent, high-quality, and real-time data streams, as shown in Figure 3.

### 3.3. Sensor Fusion Strategy

A dedicated strategy is needed to fuse the sample data produced by our heterogeneous sensors, which operate at different rates, formats, and levels of precision, to create a harmonised dataset for nowcasting. By doing this, we aim to improve the accuracy and robustness of the smart application (e.g., assisted manoeuvring). The strategy we adopt in this work is mid-level fusion, also known as feature-level fusion. This strategy helps us to capture the intricate relationship between data from different sensors. The selected features from the combined dataset will be used to nowcast wind targets measured by the meteorological station in our case. Combining environmental and operational data into a single context helps us achieve our goal with high precision and accuracy.

#### 3.3.1. Early, Middle, and Late Fusion

The most common strategies for sensor fusion are three: early fusion, middle fusion, and late fusion [15]. We can implement sensor fusion at different stages of a pipeline. Early fusion, or low-level fusion, is a strategy in which raw data from multiple sensors are fused at the lowest level. In this strategy, all information from all sensors is retained, enabling models such as deep neural networks to learn correlations among them. However, this strategy has a high computational demand, and with large enough features, we might run into the curse of dimensionality.

The second strategy to fuse sensor data is the middle-level fusion approach. This strategy is also known as feature-level fusion and lies between early-level and late-level fusion. In this strategy, sensor features are extracted and then fused. Unlike early-level fusion, we reduce dimensionality while retaining critical spatial and temporal information.

Contrary to early fusion, in late fusion or high-level fusion, each dataset from a sensor is used to train a model. When we have multiple models built specifically for a particular dataset, we use sensor combinations to perform detection or prediction. This combination enhances the system’s robustness, reliability, and accuracy. Even if one sensor malfunctions, other sensors, such as the meteorological sensor and the anemometer, provide redundancy that the system can rely upon.

#### 3.3.2. Data Preprocessing, Quality, and Fusion

A systematic preprocessing pipeline was used before processing data for modelling. By doing so, we ensure the consistency, reliability, and alignment of all the data sources.

**Figure 4:**
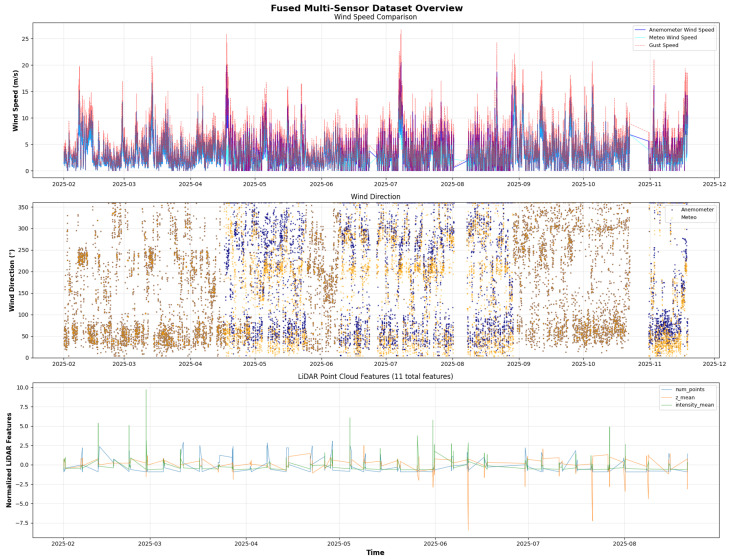
Meteorological, Anemometer and LiDAR Data.

Data Acquisition and Inspection:

As discussed in Section 3.1.1 and Section 3.1.2, data were collected from various sources, both environmental and operational. The first environmental data source is the meteorological station. In this case, the data consisted of 61,604 rows and 5 columns. As shown in Figure 4, there are short periods during which readings were missing due to the devices’ malfunctions.

The second environmental data source is the anemometer sensor. We collected raw data from two anemometers. Following the same quality checks procedures as for the previous dataset, we cleaned each anemometer dataset and combined them into a single anemometer dataset. In Figure 4, the distribution of some features over the considered time window is shown.

From the operational data, we first extracted attributes from the LiDAR files (with *.las* extension), such as the num_points (indicating the density and quality of points captured in a scan), which helps to model the environment more accurately; the means of *x*, *y*, and *z* (the 3D coordinates of each LiDAR point: east to west, north to south, and height); the minimum and maximum *z* values; the standard deviation of *z*; and other related parameters.

All the data were imported using Pandas DataFrames for the inspection, cleaning and preparation.

Missing Data Treatment:

To handle missing or incomplete data while fusing our raw sensor data, we performed cross-filling of missing values between the meteorological and anemometer sensors. The second phase, implemented after the fusion, was temporal interpolation: we resampled the fused dataset to uniform 3-min intervals using linear interpolation, with 60-min limits to fill temporal gaps.

Timestamp Standardization:

To ensure overall data consistency, we converted the timestamp fields to datetime format and set them as the DataFrame index. The interval between rows was 1 min. However, the meteorological sensor publishes data every 10 min, while the anemometer sensor publishes data every 15 min. Due to this imbalance, a time-based interpolation was performed.

Data Fusion:

We implemented a bidirectional sensor-fusion strategy to integrate data from meteorological stations, anemometers, and LiDAR systems. The fusion process employed a two-stage merge-as-of approach with a 1-h temporal tolerance: first, a meteorological-centric merge preserved all meteo timestamps while incorporating nearest anemometer measurements; second, an anemometer-centric merge retained all anemometer timestamps with corresponding meteo data. These complementary merges were concatenated and deduplicated to maximise temporal coverage. LiDAR point-cloud features were subsequently integrated using the same temporal-alignment strategy. To address missing values, we applied cross-filling, where measurements from one sensor type were used to fill gaps in another, leveraging multi-sensor redundancy. The fused dataset was then resampled to uniform 3-min intervals with linear interpolation (up to 60 min) to enhance temporal resolution, ultimately producing 128,186 samples with complete coverage of critical meteorological variables for maritime port nowcasting.

Our nowcasting task predicts three critical maritime operational parameters simultaneously: gust speed (gust_speed), wind speed (wind_speed_anemo), and wind direction (wind_direction). These targets represent the next-timestep values

$$\{{\mathrm{gust}\_\mathrm{speed}}_{t+1},\;{\mathrm{wind}\_\mathrm{speed}\_\mathrm{anemo}}_{t+1},\;{\mathrm{wind}\_\mathrm{direction}}_{t+1}\},$$

forming a multi-output prediction problem. Gust speed is the primary target for port safety assessment, as sudden gusts pose the greatest risk to crane operations and vessel handling. Wind speed provides general atmospheric conditions for operational planning, while wind direction is essential for navigation and berthing decisions. This multi-target formulation captures the interdependencies between wind intensity, gust behaviour, and directional changes, enabling comprehensive maritime safety nowcasting within a unified predictive model.

Data Normalisation and Scaling:

The StandardScaler algorithm (Equation (1)) scales to zero mean and a unit variance, as defined by:

(1) $$z=\frac{x-\mu }{\sigma }$$

where *x* is the starting value, μ is the mean value, and σ is one standard deviation of a feature was utilised. All input features and prediction targets were standardised using z-score normalisation (Equation (1)) to ensure stable training across models with different scale requirements. The 46 numeric input features, which included measurements with varying units and scales (e.g., atmospheric pressure ∼1013hPa, temperature ∼$18^{\;\circ }C$, wind speed ∼3–4m/s), were transformed. The three prediction targets (gust speed, wind speed, and wind direction) were similarly normalised. To prevent data leakage, the scaler was fitted exclusively on the training set (70% of data), with the learnt parameters then applied to transform the validation (15%) and test sets (15%). Separate scalers were maintained for features (scaler_X) and targets (scaler_y), enabling inverse transformation of predictions back to the original units for interpretability and operational use. This standardisation approach is essential for neural network convergence, ensures features contribute equally regardless of their native scales, and accelerates gradient descent during training.

### 3.4. Machine Learning Models

Real-time nowcasting in seaport environments requires ML/AI models capable of handling temporally correlated sensor data while operating reliably with limited historical data and rapidly changing conditions. We employed ensemble methods, recurrent neural networks, convolution-recurrent hybrids, probabilistic deep learning, and attention-based architectures to predict short-term wind targetswithin port areas. These models were selected to capture both the nonlinear relationships and temporal dynamics inherent in the data. The models evaluated in this work each bring different strengths aligned with the operational constraints mentioned above in seaports.

#### 3.4.1. Random Forest

Random Forest (RF) [16] is an ensemble learning method that constructs multiple decision trees using bootstrap samples and random feature subsets. The final prediction is obtained by averaging across all trees, providing robustness against overfitting and variance.

#### 3.4.2. XGBoost

Extreme Gradient Boosting (XGBoost) [17] is a scalable tree-based ensemble technique that builds additive models sequentially, where each tree corrects the residuals of the previous ones. Regularisation and efficient parallelisation make it suitable for structured data regression tasks.

#### 3.4.3. Kalman Filter

The Kalman filter [18] is a recursive state-space estimation algorithm that provides optimal predictions for linear dynamical systems under Gaussian noise assumptions. At each timestep, it combines a prior state estimate with new sensor observations to produce a posterior estimate with reduced uncertainty, making it well-suited for smoothing and forecasting noisy meteorological signals. Its sequential update structure allows efficient real-time inference, which is valuable for port-scale environmental monitoring where measurements from heterogeneous sensors arrive asynchronously.

#### 3.4.4. Long Short-Term Memory (LSTM)

Long Short-Term Memory networks [19] extend recurrent neural networks by incorporating gating mechanisms that allow them to retain long-term dependencies. This makes them particularly effective for modelling temporal sequences, such as wind speed data.

#### 3.4.5. 1D Convolutional Neural Network with Temporal Convolutional Network (1D-CNN/TCN))

The 1D-CNN/TCN architecture [20] combines dilated causal convolutions with residual connections to capture multi-scale temporal patterns in time-series data. Unlike ConvLSTM (designed for spatiotemporal data with 2D/3D spatial structure), TCN operates directly on 1D temporal sequences, making it well-suited for tabular sensor time series. Exponentially increasing dilation rates (1, 2, 4, 8) to enable the network to capture both short-term fluctuations and long-term atmospheric dynamics with fewer parameters.

#### 3.4.6. Ensemble Neural Network

Ensemble Neural Networks [21] combine predictions from multiple independently trained feedforward networks with varying architectures and dropout rates. The ensemble approach provides uncertainty quantification through model disagreement (standard deviation across predictions).

#### 3.4.7. Transformer

Transformers [22] leverage self-attention mechanisms to capture long-range dependencies in sequences without relying on recurrence. Their parallel computation and scalability make them powerful for complex temporal modelling tasks.

#### 3.4.8. Training Parameters

The hyperparameters for each model, set to balance complexity and performance, are shown in the Table 1 below.

### 3.5. Experimental Setup

Details of the data split, evaluation metrics, and system setup are provided below.

#### 3.5.1. Train/Validation/Test Split

To prevent look-ahead bias, we applied a chronological split (no shuffling): 70% of the samples were used as the training set, 15% for validation, and the remaining 15% for testing. For cross-validation, time-series-aware folds (rolling/expanding windows) were employed instead of random shuffling.

**Traditional ML models:** XGBoost, Random Forest, and Kalman Filter each use **46 engineered features** from the current timestep as input.

**Deep learning models:** LSTM, Transformer, 1D-CNN/TCN, and Ensemble NN. Each sample uses **12 historical timesteps** as a lookback window. Each has 46 features.

**Cross-validation:** Deep learning models use an extended sequence length of **24 timesteps** to improve temporal pattern learning.

All models predict the next timestep (1-step-ahead forecast) for three target variables simultaneously (gust speed, wind speed, and wind direction) in a multi-output regression framework, with features and targets normalised independently using StandardScaler.

#### 3.5.2. Evaluation Metrics

Model performance was assessed using root mean squared error (RMSE), mean absolute error (MAE), and the coefficient of determination ($R^{2}$):

$$\mathrm{RMSE}=\sqrt{\frac{1}{N}\sum_{i=1}^{N}{\left(y_{i}-{\hat{y}}_{i}\right)}^{2}},\;\mathrm{MAE}=\frac{1}{N}\sum_{i=1}^{N}\left|y_{i}-{\hat{y}}_{i}\right|,\;R^{2}=1-\frac{\sum_{i=1}^{N}{\left(y_{i}-{\hat{y}}_{i}\right)}^{2}}{\sum_{i=1}^{N}{\left(y_{i}-\bar{y}\right)}^{2}}.$$

#### 3.5.3. Hardware and Software

All models were trained on a workstation with a 6-core (12-thread) Intel CPU (x86_64), 16 GB RAM, and an NVIDIA GeForce RTX 2060 GPU. Experiments were implemented in Python 3.12.3 using PyTorch 2.8.0+cu128, scikit-learn 1.7.1, and CUDA 12.8 for GPU acceleration.

## 4. Results

In this section, we present key observations from the experiments, including overall model performance and generalisation, model-specific performance analysis, and target-specific performance patterns.

### 4.1. Overall Model Performance and Generalisation

Across all seven evaluated models, XGBoost achieved the highest predictive accuracy and the most stable generalisation (see Table 2: Test Set Performance and Table 3: Cross-Validation Performance). Test-set performance was near-perfect for all three targets (R^2^ = 0.9991), and the cross-validation score remained equally strong (CV R^2^ = 0.9976), producing a negligible generalisation gap (Δ=0.0015, see Table 4: Test vs CV Comparison). This exceptional stability indicates minimal overfitting and robust performance across diverse temporal conditions.

The Ensemble Neural Network (5-model ensemble) achieved the second-best test performance (R^2^ = 0.9633), representing a substantial improvement over traditional Bayesian approaches. Its cross-validation performance (CV R^2^ = 0.8231) reveals a moderate generalisation gap (Δ=0.1402), suggesting some sensitivity to training-fold variability. Despite this gap, the Ensemble NN’s ability to provide fast uncertainty quantification (20× faster than MC Dropout) makes it valuable for safety-critical applications.

Random Forest and Kalman Filter demonstrated strong performance (Test R^2^ = 0.9614 and 0.9190, respectively) with acceptable stability (Δ=0.0559 and Δ=0.0256). The Kalman Filter’s particularly small generalisation gap reflects its model-based approach, which avoids overfitting to training patterns through recursive Bayesian estimation rather than parameter fitting.

The 1D-CNN (Temporal Convolutional Network) achieved solid performance (Test R^2^ = 0.9384, CV R^2^ = 0.8675, Δ=0.0708), demonstrating proper time-series architecture. This highlights the importance of architecture–data alignment: TCN’s 1D convolutional layers are purpose-built for time series, effectively capturing multi-scale temporal patterns.

LSTM showed reasonable test performance (R^2^ = 0.8611) with cross-validation accuracy (CV R^2^ = 0.8477, Δ=0.0134), indicating stable generalisation ability but lower overall accuracy. Transformer (Test R^2^ = 0.8331, CV R^2^ = 0.7849, Δ=0.0482) exhibited the weakest performance among deep learning models, likely due to insufficient training data for its complex attention mechanism.

The findings indicate that for tabular time-series wind data, tree-based methods, particularly gradient boosting, provide the best balance of accuracy and reliability. Well-designed modern neural architectures can also deliver results comparable to those of traditional architectures. However, ensemble neural networks, 1D-CNNs, and TCNs require careful architecture selection and ample training data.

**Figure 5:**
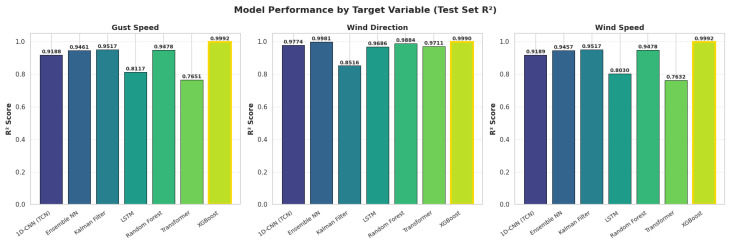
Models Test-set Performance.

**Figure 6:**
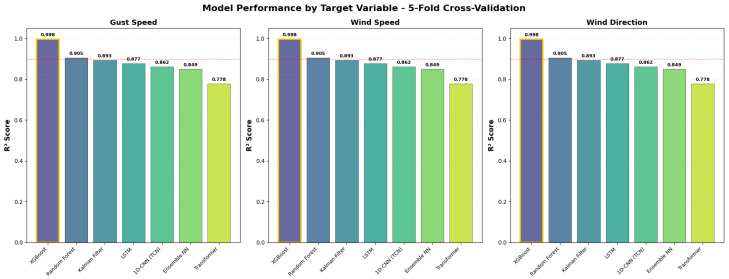
ModelsCross-Validation Performance.

**Figure 7:**
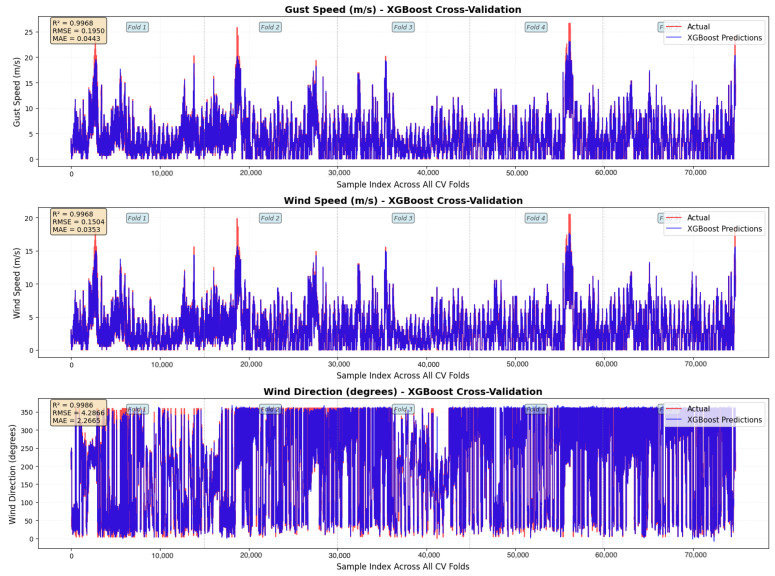
Best Performing Model.

**Key Findings** (see Figure 5, Figure 6 and Figure 7 for visual comparison):

- LSTM: Δ=0.0134 (generalises fairly good, moderate accuracy)
- XGBoost: Δ=0.0015 (exceptional generalisation)
- Kalman Filter: Δ=0.0256 (model-based robustness)
- Transformer: Δ=0.0482 (attention mechanism, lowest DL accuracy)
- Random Forest: Δ=0.0559 (good stability)
- 1D-CNN/TCN: Δ=0.0708 (proper time-series architecture)
- Ensemble NN: Δ=0.1402 (moderate, but second-best test accuracy)

The cross-validation results can be seen in Table 3. The standard ensemble schemes (XGBoost, Random Forest) showed very stable performance across folds. The Ensemble Neural Network also demonstrated strong performance, providing evidence in favour of uncertainty-aware modelling.

### 4.2. Ensemble Neural Network

The Ensemble Neural Network achieved exceptional test-set accuracy (R^2^ = 0.9633), ranking second only to XGBoost among all evaluated models. This architecture replaces the previous Bayesian Neural Network with Monte Carlo Dropout, delivering critical improvements in speed and uncertainty quantification:

The ensemble consists of five independent neural networks, each with a [512, 256, 128] hidden-layer architecture, batch normalisation, and conservative dropout (0.2). Models are trained independently with different random initialisations, creating diversity through weight-space exploration rather than stochastic dropout.

**Uncertainty Quantification via Model Disagreement:** Unlike MC Dropout’s aleatoric uncertainty (which captures data noise), the ensemble provides epistemic uncertainty (which captures model knowledge gaps). When predictions from the five models diverge, the system signals low confidence, critical for maritime safety decisions such as:

- crane operations near wind-speed thresholds (12–15 m/s),
- vessel berthing during marginal conditions,
- emergency response planning during storm approach.

The moderate generalisation gap (Δ=0.1402) is acceptable given the ensemble’s practical advantages. Cross-validation performance (CV R^2^ = 0.8231) remains strong, demonstrating robust performance across temporal folds. The gap likely stems from the ensemble’s higher capacity (5 models × 650 k parameters each), which can memorise training patterns but still generalises adequately.

### 4.3. Temporal Convolutional Network

The Temporal Convolutional Network (1D-CNN) achieved strong performance (Test R^2^ = 0.9384, CV R^2^ = 0.8675), demonstrating the effectiveness of proper time-series architecture. The model shows good generalisation (Δ=0.0708) and ranks third among all models, highlighting the importance of matching architecture to data structure.

The moderate generalisation gap (Δ=0.0708) is satisfactory, indicating that parallel convolutional processing generalises efficiently on this dataset. This stability, combined with strong test performance, makes TCN a viable alternative to ensemble methods when interpretability is less critical.

### 4.4. Target-Specific Performance Patterns

Performance varied across the three target variables, gust speed, sustained wind speed, and wind direction, revealing distinct model strengths.

**Wind speed and gust speed.** Tree-based models delivered uniformly strong performance for both speed-related targets, with XGBoost achieving $R^{2}\approx 0.999$ for both gust and wind speed. Random Forest and the Kalman Filter also performed well. Neural networks generally struggled with these variables, likely due to the complex, nonlinear dependencies underlying wind dynamics, including temperature gradients, atmospheric stability, and turbulence intensity. Gradient boosting was particularly effective at capturing these interactions through iterative residual learning.

**Wind direction.** Most models achieved higher accuracy for wind direction than for wind speed. Recurrent and attention-based architectures, including LSTM, Transformer, and Bayesian NN, performed notably well ($R^{2}$ between 0.966 and 0.999), reflecting the strong temporal persistence inherent in wind direction. However, XGBoost still achieved the highest performance ($R^{2}=0.9990$), indicating that lag-based feature engineering can replicate the memory effects captured by deep learning.

**Kalman Filter inconsistency.** While the Kalman Filter achieved strong predictive performance for wind speed, it performed substantially worse for wind direction. This arises from the circular nature of directional data, which violates the linear Gaussian assumptions of standard Kalman filtering. Although sine/cosine encoding partially mitigates this issue, wind direction often exhibits abrupt shifts and non-Gaussian distributions, suggesting that more advanced circular state-space models would be more appropriate.

### 4.5. Summary of Key Findings

XGBoost consistently outperforms all other models in both accuracy and generalisation, achieving near-perfect predictions across all targets.

Ensemble NN delivers competitive performance (2nd-best R^2^) with fast uncertainty quantification, demonstrating strong accuracy in both speed and direction.

1D-CNN/TCN is the best deep learning architecture (3rd overall), demonstrating that properly designed temporal convolutions can effectively model maritime wind patterns.

LSTM shows good generalisation with moderate absolute accuracy, demonstrating stable performance without significant overfitting.

Wind direction is easier to model than wind speed, as temporal continuity dominates its dynamics, benefiting both tree-based and recurrent models.

## 5. Discussion

This work demonstrates that a oneM2M-enabled sensor fusion pipeline can effectively support short-term meteorological nowcasting in port environments. Across all evaluated models, XGBoost delivered the most accurate and consistent performance, achieving near-perfect results (Test $R^{2}\approx 0.999$; CV $R^{2}\approx 0.9976$) with an extremely small generalisation gap. This confirms that tree-based ensemble models are highly suitable for heterogeneous, tabular sensor-fusion data, especially when the available historical window is limited.

Deep learning models displayed more modest performance. The 1D-CNN/TCN achieved strong and stable results, confirming the value of temporal convolutions for short-horizon patterns. Conversely, LSTM and Transformer underperformed relative to XGBoost due to limited data history and the simplicity of the one-step forecasting task. The Ensemble Neural Network showed high accuracy and fast uncertainty estimation, making it a practical candidate for safety-critical decision support even though its generalisation gap was larger.

Target-specific patterns reveal that wind direction is easier to predict due to its temporal persistence, while gust speed, the most safety-critical variable, is highly nonlinear yet effectively modelled by XGBoost. The Kalman Filter performed well for wind speed but poorly for direction, highlighting its limitations with circular variables.

Overall, results indicate that ensemble and probabilistic approaches outperform deep architectures for real-time port nowcasting, offering robustness, speed, and reliability.

### Limitations

Although the proposed framework demonstrates strong predictive performance and robustness across heterogeneous sensor streams, several factors constrain the generality and scalability of the current results. These limitations primarily stem from the characteristics of the available dataset, the operational constraints of the oneM2M infrastructure, and the specific conditions under which the system was deployed.

**Key limitations include:**

- **Limited dataset duration** (∼10 months), derived from the most recent operational trials at the Port of Livorno. This restricts the model’s exposure to longer-term seasonal behaviours and infrequent extreme events.
- **Short-term nature of oneM2M storage**: the oneM2M platform is not designed for long-term data retention; it functions instead as a real-time store-and-share system.
- **Sensor outages**: periods of missing measurements occurred despite mitigation efforts through cross-filling and interpolation.
- **Limited LiDAR operational diversity**: all LiDAR data originated from a single vessel and a narrow set of manoeuvre scenarios, potentially reducing generalisability to other vessel types or port configurations.

## 6. Conclusions and Future Works

This work presents a sensor fusion-based machine learning framework for nowcasting gust speed, wind speed, and wind direction in port environments. By integrating heterogeneous environmental and operational data through a oneM2M-compliant architecture, the system provides high-quality, synchronised inputs for prediction models.

Among all tested algorithms, XGBoost clearly emerged as the best performer, achieving near-perfect accuracy and exceptional generalisation. Ensemble NN and TCN also showed strong performance, while deep recurrent and transformer architectures were limited by dataset scale.

These findings demonstrate that gradient boosting and ensemble methods are highly effective for real-time maritime nowcasting, supporting safer manoeuvring, improved berthing operations, and enhanced situational awareness for MASS and port digital twin platforms. In the future, we will address the following topics to further enhance sensor fusion techniques and models.

- Multi-step forecasting (10–30 min ahead) for operational planning.
- Additional sensor modalities (radars, AIS, GNSS, cameras) to enrich fusion.
- Cross-port training for improved generalisation and transferability.
- Real-time deployment with online learning, drift detection, and continuous updates.

## Figures and Tables

**Table 1:** Summary of models and key hyperparameters (see Appendix A for full configurations).

Model	Key Hyperparameters (Summary)
Random Forest	200 trees; max depth 15; min split 5
XGBoost	200 estimators; depth 8; learning rate 0.05
Kalman Filter	Constant-velocity model; Q = 0.01; R = 0.1
LSTM (BiLSTM + Attention)	2 layers; hidden size 128; seq = 12; dropout 0.2
1D-CNN (TCN)	3 layers; 64 filters; kernel size 3; dilation [1, 2, 4]
Ensemble NN	5 models; layers 384–192–96; dropout 0.3
Transformer	4 layers; $d_{model}=128$; 8 heads; FF = 512

**Table 2:** Model performance Test Set.

Target	Model	R^2^	MAE	RMSE
gust_speed	1D-CNN (TCN)	**0.918847**	0.695039	0.987058
	Ensemble NN	**0.946117**	0.604951	0.804465
	Kalman Filter	**0.951658**	0.488189	0.761976
	LSTM	0.811729	1.133083	1.503423
	Random Forest	**0.947846**	0.516235	0.791450
	Transformer	0.765102	1.220314	1.679307
	XGBoost	**0.999208**	0.030492	0.097508
wind_direction	1D-CNN (TCN)	**0.977369**	8.405764	16.497965
	Ensemble NN	**0.998084**	1.457373	4.800798
	Kalman Filter	0.851620	23.789816	42.243449
	LSTM	**0.968588**	7.600652	3.436830
	Random Forest	**0.988389**	7.963296	11.816804
	Transformer	**0.971085**	9.334155	18.648239
	XGBoost	**0.999032**	1.769567	3.412293
wind_speed_anemo	1D-CNN (TCN)	**0.918919**	0.537593	0.758940
	Ensemble NN	**0.945746**	0.466099	0.620946
	Kalman Filter	**0.951658**	0.375530	0.586135
	LSTM	0.802976	0.892646	1.183057
	Random Forest	**0.947846**	0.397104	0.608807
	Transformer	0.763152	0.941695	1.297125
	XGBoost	**0.999205**	0.024204	0.075182

**Table 3:** Cross-validation performance per target (5 folds).

Target	Model	R^2^	MAE	RMSE
gust_speed	1D-CNN (TCN)	0.867540	4.727989	9.861423
	Ensemble NN	0.823072	5.657557	10.135465
	Kalman Filter	0.893374	6.870698	13.283340
	LSTM	0.798000	1.110000	1.540000
	Random Forest	**0.905442**	4.575846	7.324737
	Transformer	0.784913	5.759604	12.364203
	XGBoost	**0.997608**	0.782044	1.493644
wind_direction	1D-CNN (TCN)	0.867540	4.727989	9.861423
	Ensemble NN	0.823072	5.657557	10.135465
	Kalman Filter	0.893374	6.870698	13.283340
	LSTM	**0.955000**	7.300000	17.900000
	Random Forest	**0.905442**	4.575846	7.324737
	Transformer	0.784913	5.759604	12.364203
	XGBoost	**0.997608**	0.782044	1.493644
wind_speed_anemo	1D-CNN (TCN)	0.867540	4.727989	9.861423
	Ensemble NN	0.823072	5.657557	10.135465
	Kalman Filter	0.893374	6.870698	13.283340
	LSTM	0.790000	0.860000	1.220000
	Random Forest	**0.905442**	4.575846	7.324737
	Transformer	0.784913	5.759604	12.364203
	XGBoost	**0.997608**	0.782044	1.493644

**Table 4:** Comparison between Test Set and Cross-Validation Performance (R^2^, averaged across all targets).

Model	Test R^2^	CV R^2^	Δ (Test − CV)
1D-CNN (TCN)	0.9384	0.8675	0.0708
Ensemble NN	0.9633	0.8231	0.1402
Kalman Filter	0.9190	0.8934	0.0256
LSTM	0.8611	0.8477	0.0134
Random Forest	0.9614	0.9054	0.0559
Transformer	0.8331	0.7849	0.0482
XGBoost	0.9991	0.9976	0.0015

## Data Availability

The data presented in this study are available on request from the corresponding author. Access to the data can be granted upon approval by the entities acknowledged in this manuscript, who hold the rights to the dataset.

## Appendix A. Model Hyperparameter Configurations

**Model**	**Hyperparameters**
Random Forest	• **n_estimators**: 200
	• **max_depth**: 15
	• **min_samples_split**: 5
	• **n_jobs**: −1
	• **random_state**: 42
	• **multi-output**: native
XGBoost	• **n_estimators**: 200 (test), 500 (final)
	• **max_depth**: 8
	• **learning_rate**: 0.05
	• **subsample**: 0.8
	• **colsample_bytree**: 0.8
	• **min_child_weight**: 3
	• **gamma**: 0.1; reg_alpha: 0.1; reg_lambda: 1.0
	• **multi-output**: MultiOutputRegressor
Kalman Filter	• **n_targets**: 3
	• **process noise**: 0.01
	• **measurement noise**: 0.1
	• **state model**: constant velocity
	• **state dimension**: 6 (3 targets × 2)
LSTM (BiLSTM + Attention)	• **architecture**: BiLSTM + Attention
	• sequence length: 12 (test), 24 (CV)
	• hidden size: 128; layers: 2
	• dropout: 0.2; batch size: 256
	• bidirectional: True
	• optimizer: AdamW (lr = 0.001, wd = $10^{-5}$)
	• scheduler: CosineAnnealingWarmRestarts $\left(T_{0}=10,T_{mult}=2\right)$
	• epochs: 60; early stopping: 15; grad clipping: 5.0
1D-CNN (TCN)	• **architecture**: Temporal Convolutional Network
	• sequence length: 12 (test), 24 (CV)
	• channels: [64, 128, 128, 256]
	• kernel size: 3; dilation: [1, 2, 4, 8]
	• dropout: 0.2; batch size: 256
	• residual connections: Yes
	• optimizer: AdamW (lr = 0.001, wd = $10^{-5}$)
	• scheduler: ReduceLROnPlateau (factor = 0.5, patience = 5)
	• epochs: 50; early stopping: 10; grad clipping: 1.0
Ensemble NN (5-model)	• **n_models**: 5
	• architecture: [512, 256, 128]
	• dropout: 0.2; batch size: 128
	• BatchNorm: Yes; weight init: Kaiming Normal
	• optimizer: AdamW (lr = 0.001, wd = $10^{-4}$)
	• scheduler: CosineAnnealingLR $\left(T_{\max}=50,\eta_{min}=10^{-6}\right)$
	• epochs: 50; early stopping: 15
	• uncertainty: ensemble disagreement (std)
Transformer	• **architecture**: Encoder-only
	• $d_{\mathrm{model}}=128$; nhead = 8; layers = 4
	• dropout: 0.2; FF dim: 512
	• sequence length: 12 (test), 24 (CV)
	• positional encoding: sinusoidal
	• batch size: 256
	• optimizer: AdamW (lr = $5\times 10^{-4}$, wd = $10^{-5}$)
	• scheduler: ReduceLROnPlateau (factor = 0.5, patience = 5)
	• epochs: 50; early stopping: 10; grad clipping: 1.0
